# The role of ^18^F-FDG PET/CT in diagnosing cardiac infiltration and therapeutic evaluation in extranodal nasal-type NK/T-cell lymphoma: a case report

**DOI:** 10.3389/fonc.2025.1548785

**Published:** 2025-03-07

**Authors:** Taiping Liao, Lingxiao Li, Guoxu Fu, Li Li, Qinlin Qi, Yongjun Long

**Affiliations:** Department of Nuclear Medicine, The Third Hospital of Mianyang(Sichuan Mental Health Center), Mianyang, China

**Keywords:** 18 F, FDG, PET/CT, NK/T-cell lymphoma, nasal

## Abstract

Extranodal nasal-type NK/T-cell lymphoma is closely associated with Epstein-Barr virus (EBV) infection and primarily involves the nasopharynx. We report a rare case of extranodal nasal-type NK/T-cell lymphoma with widespread lymphomatous infiltration, including cardiac involvement, an exceedingly uncommon manifestation. This case emphasizes the pivotal role of^18^F-FDG PET/CT in diagnosing and evaluating treatment outcomes in extranodal nasal-type NK/T-cell lymphoma

## Introduction

Extranodal nasal-type NK/T-cell lymphoma predominantly occurs in the nasal cavity and upper aerodigestive tract (80%), making it one of the major pathological subtypes. While the primary sites are typically the nasal cavity and nasopharynx, extranodal NK/T-cell lymphoma may occasionally involve the gastrointestinal tract, skin, testes, and other locations. We report a rare case of extranodal nasal-type NK/T-cell lymphoma confirmed by nasopharyngeal biopsy, where ^18^F-FDG PET/CT revealed cardiac infiltration during staging and was further used for therapeutic evaluation post-treatment.

## Case presentation

A 33-year-old male presented with a 3-year history of recurrent nasal obstruction, significantly worsening 10 days prior to admission. CT and MRI revealed a mass in the right nasal cavity, suspected to be a tumor. A biopsy confirmed extranodal nasal-type NK/T-cell lymphoma with the following immunohistochemistry (IHC) results: CD3(+), CD4(+), CD8(+), CD56(+), granzyme B(+), CD20 (–), pan-CK (–), SOX10 (–), and Ki-67 (50%). EBV testing was positive.

**Figure 1:**
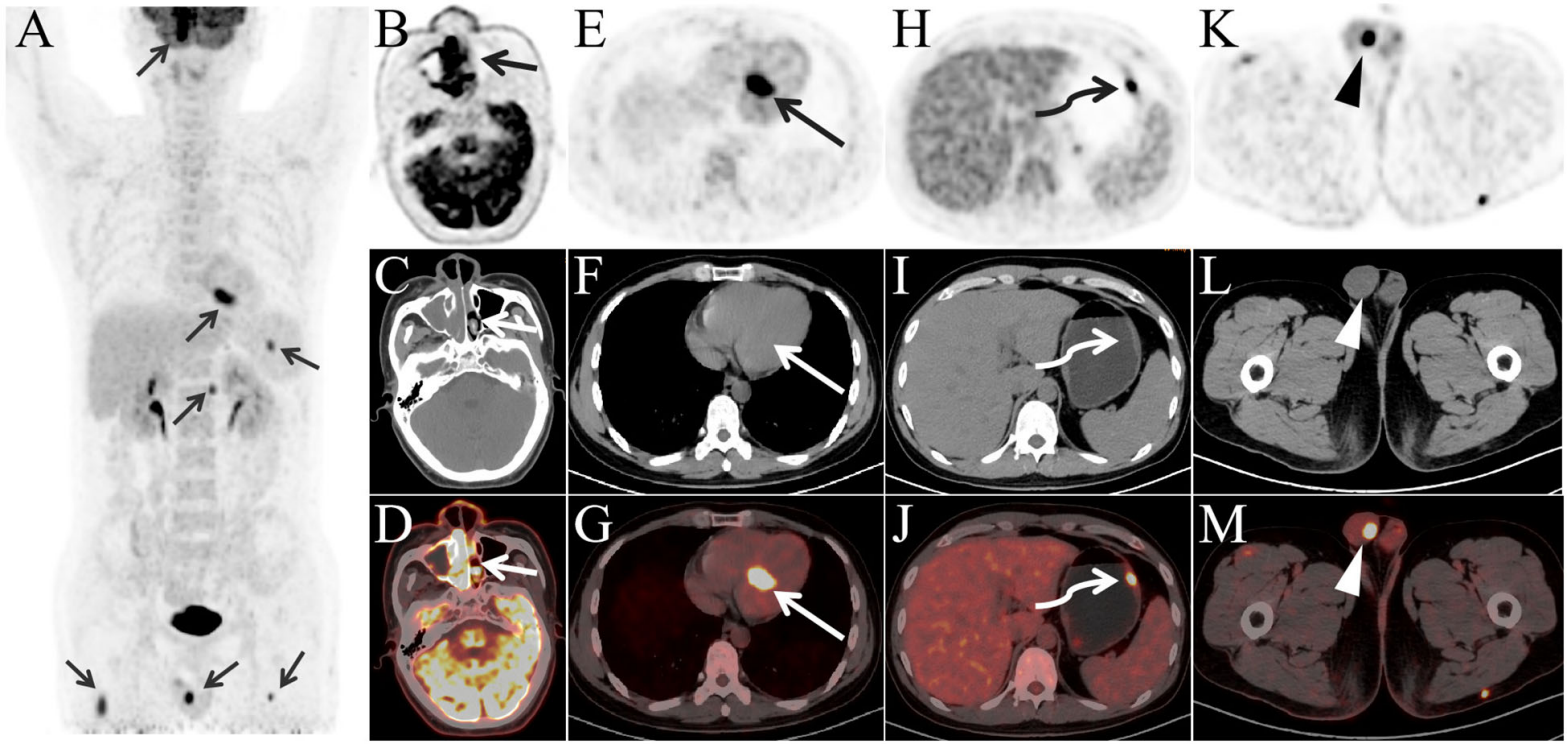
The maximum intensity projection image **(A)** reveals multiple FDG-avid lesions (arrow) throughout the body. Panels **(B–D)** depict soft tissue in the bilateral nasal cavity and right maxillary sinus (short arrow) with significantly increased FDG uptake, SUVmax 16.9. Panels **(E–G)** show an FDG-avid lesion in the left atrium (long arrow), SUVmax 14.6. Panels **(H–J)** demonstrate a mildly thickened gastric wall (curved arrow) with elevated FDG uptake, SUVmax 9.3, while panels **(K–M)** reveal a nodular FDG-avid lesion in the right testis (arrowhead), SUVmax 9.3.

For precise staging, ^18^F-FDG PET/CT was performed. The patient fasted for 10 hours before receiving an injection of 7.0 mCi FDG. The patient rested for 50 minutes after receiving the FDG injection, and then PET/CT imaging was performed using a United Imaging uMI 550 scanner. The SUV values were calculated based on the patient’s Body Mass Index (BMI) using the formula:


$$\text{SUV}=\frac{\text{Radioactivity concentration of the ROI (Bq/g)}}{\text{ Injected radioactivity}(\text{Bq})/{\text{BMI (kg/m}}^{2})}/\text{k}$$

where ROI represents the region of interest, and k is the radionuclide decay correction factor. PET/CT (**Figures 1A–M**) revealed lymphoma lesions in the bilateral nasal cavity and right maxillary sinus (**Figures 1B–D**), the left atrium (**Figures 1E–G**), gastric body (**Figures 1H–J**), and right testis (**Figures 1K–M**). Additional lesions were found in the skin and muscles, but are not shown in the images. The patient was staged as Lugano stage IV with a prognostic score of NRI: 3.

**Figure 2:**
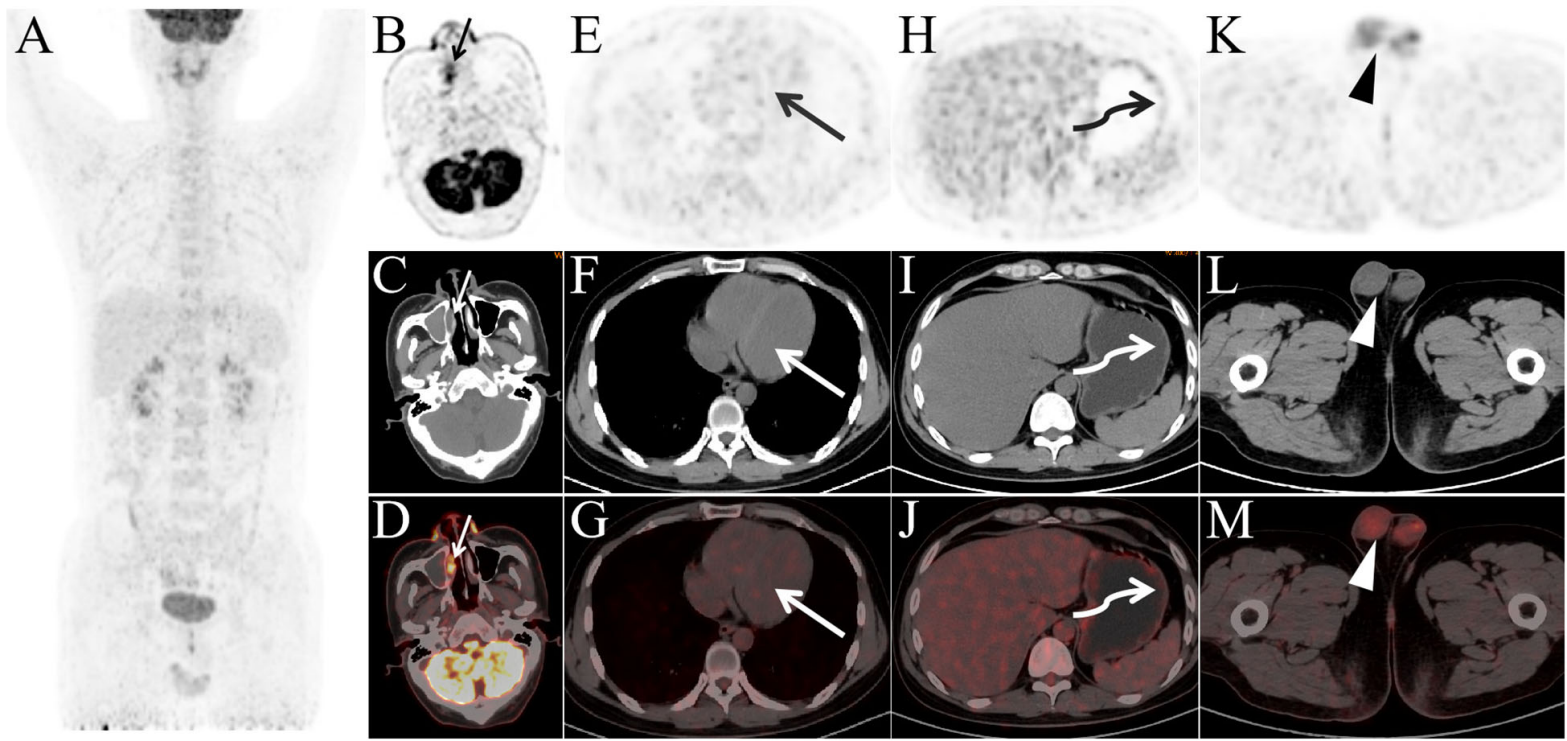
Panels **(A–M)** present the post-treatment PET/CT images after two cycles of therapy. Panel **(A)** shows that, compared to the pre-treatment images, FDG-avid lesions have nearly disappeared throughout the body. Panels **(B–D)** show a slight reduction in the soft tissue of the bilateral nasal cavity and right maxillary sinus, with a significant decrease in FDG uptake, SUVmax 6.3. The previously observed FDG-avid lesions in the left atrium (long arrow, SUVmax 1.0), gastric body (curved arrow, SUVmax 1.3), and right testis (arrowhead, SUVmax 2.0) no longer exhibit elevated FDG metabolism.

The patient underwent a total of four cycles of SMILE chemotherapy, with no treatment delays or interruptions during the course of therapy. Twenty days after the completion of the second cycle, a follow-up ^18^F-FDG PET/CT (**Figures 2A–M**) showed significant reductions in FDG uptake across previously detected lesions. The Deauville score for the nasal cavity and right maxillary sinus lesions was 5, while the score for the other lesions was 1, indicating a partial response (PR).

## Discussion

Extranodal nasal-type NK/T-cell lymphoma is predominantly observed in Asia and South America, with rarity in other regions (1). Unlike other lymphomas, NK/T-cell lymphoma mainly arises in extranodal sites, with nasal-type accounting for 80% of cases (2).SMILE chemotherapy is an effective treatment for newly diagnosed stage IV, relapsed or refractory extranodal nasal-type NK/T-cell lymphoma (3).

PET/CT is the standard imaging modality for extranodal NK/T-cell lymphoma, outperforming CT and MRI in detecting systemic lymphoma lesions,it enables earlier and more comprehensive detection of lymphoma lesions (4, 5). In newly diagnosed patients, ^18^F-FDG PET/CT provides precise staging, essential for therapeutic decision-making (6, 7). It also plays a critical role in assessing therapeutic response, as interim and post-treatment PET/CT findings are predictive of prognosis (8–10). Parameters such as TLG, SUVmax, and SUVmean are key prognostic indicators for extranodal NK/T-cell lymphoma (11).

In this case, ^18^F-FDG PET/CT revealed rare cardiac infiltration with high metabolic activity, which resolved following treatment. Reports of ^18^F-FDG PET/CT identifying cardiac infiltration in extranodal nasal-type NK/T-cell lymphoma are rare. Our study emphasizes the importance of ^18^F-FDG PET/CT in staging and therapeutic evaluation of extranodal nasal-type NK/T-cell lymphoma, offering valuable insights for its diagnosis and management.

Based on the findings in this case, PET/CT should be the preferred imaging modality for staging extranodal NK/T-cell lymphoma. It has superior sensitivity in detecting systemic involvement, including rare cardiac infiltration. For follow-up, regular PET/CT scans are essential to monitor recurrence. If PET/CT is unavailable, MRI or echocardiography may help assess cardiac involvement. In NK/T-cell lymphoma with cardiac infiltration, special therapeutic considerations are needed. Cardiac involvement may increase the risk of arrhythmias and heart failure. A multidisciplinary team of hematologists, oncologists, and cardiologists is crucial. Close cardiac monitoring, including ECG and echocardiography during treatment, is essential. Treatment regimens should balance efficacy with potential cardiotoxicity.

After 3 years of imaging follow-up, the patient remains in complete remission (CR) without any discomfort.

## Data Availability

The original contributions presented in the study are included in the article/supplementary material. Further inquiries can be directed to the corresponding author.
